# Socioeconomic inequality and determinants of postnatal home visits made by public health midwives: An analysis of the Sri Lanka Demographic and Health Survey

**DOI:** 10.1371/journal.pone.0215816

**Published:** 2019-04-24

**Authors:** Dhammika Deepani Siriwardhana, Arunasalam Pathmeswaran, Ananda Rajitha Wickremasinghe

**Affiliations:** 1 Research Department of Primary Care and Population Health, University College London, Rowland Hill Street, London, United Kingdom; 2 Department of Disability Studies, Faculty of Medicine, University of Kelaniya, Ragama, Sri Lanka; 3 Department of Public Health, Faculty of Medicine, University of Kelaniya, Ragama, Sri Lanka; University of South Carolina Arnold School of Public Health, UNITED STATES

## Abstract

**Introduction:**

The impact of socioeconomic inequalities on health outcomes and service delivery is increasingly researched globally. This study assessed the overall and sector-wise socioeconomic inequality in postnatal home visits made by Public Health Midwives (PHMs) in Sri Lanka and decomposed the observed socioeconomic inequality into potential determinants.

**Methods:**

Data from the Sri Lanka Demographic and Health Survey (SLDHS) 2006–07 were used. Data were collected from ever-married women who gave birth to their last child in 2001 or later (up to 2007). Whether the PHM visited the home to provide postnatal care within one month of the delivery was the health outcome of interest. Sri Lanka is divided into three sectors (areas) as urban, rural, and estate (plantation) based on the geographical location and the availability of infrastructure facilities. Concentration indices were calculated and concentration curves were plotted to quantify the overall and sector-wise socioeconomic inequality. Decomposition analysis using probit regression was performed to estimate the contribution of potential determinants to the observed socioeconomic inequality.

**Results:**

Overall, 83.0% of women were visited by a PHM within one month of the delivery. The highest number of home visits was reported in the rural sector (84.5%) and lowest was reported from the estate sector (72.4%). A pro-poor, pro-rich, and no inequality were observed across urban, rural, and estate sectors respectively. Wealth had a small contribution to the estimated inequality. Province of residence and the education level of women were the main determinants of the observed socioeconomic inequality.

**Conclusion:**

Addressing the socioeconomic inequality of postnatal home visits made by PHMs should not be seen as a health system issue alone. The associated social determinants of health should be addressed through a multi-sectoral approach encompassing the principles of primary health care.

## Introduction

Moving from the Millennium Development Goals, maternal and child health is emphasized in Sustainable Development Goal (SDG) 3 under the global rubric of good health and wellbeing. Two targets related to maternal and child health are included in SDG 3; to reduce the global maternal mortality ratio to less than 70 per 100,000 live births and to end preventable deaths of newborns and children under five years of age. In addition, the SDGs also address the social determinants of health focusing on poverty, education, gender equality and reducing inequalities [1]. Health inequalities exist within and between countries. A health inequality becomes a health inequity when it is unjust and unfair [2]. Therefore, quantifying these inequalities using appropriate measures is a major step in understanding them [3].

Sri Lanka, with a population of about 21 million, is a lower-middle income country with a per capita gross domestic product of USD 3857 in 2016 and a high human development index of 0.766 [4]. The Sri Lankan health system has been viewed as a model for other developing countries[5] for its outstanding performances in the areas of maternal and child health at low cost. The maternal mortality ratio, neonatal mortality rate, and under five mortality rate in Sri Lanka were reported to be 33.8 per 100,000 live births, 5.8 per 1000 live births, and 9.3 per 1000 live births respectively in 2016 [6]. Despite these tremendous achievements, health inequalities are often observed between socioeconomic strata, education levels, and geographical regions [7].

Postnatal care is important in reducing maternal and neonatal deaths. It provides a supportive environment to the mother, the newborn baby, and the wider family [8]. Postnatal care in Sri Lanka is provided through hospitals, field clinics, and home visits. In Sri Lanka, almost all births take place at a hospital [9]. Following discharge from a hospital, the mother and baby are cared for at field clinics run by a medical officer with support staff and through postnatal home visits made by field public health midwives [10, 11].

The field public health midwife plays a critical role in providing home-based care. Every household in Sri Lanka is listed under a defined PHM area which serves a population of 3000–5000. The PHM is a well-trained government maternal and child health care provider at field level; she is expected to visit a postnatal mother at least twice during the first 10 postnatal days, the first of which should be done within 5 days, if the delivery is an uncomplicated vaginal delivery. The PHM is also expected to make a home visit between 11–28 days after delivery and another one around 42 days after delivery if other complications are not detected earlier [12]. The PHM provides information on danger signs to the mother and the timely steps to be taken in case of a problem. She also supports and promotes breast feeding, immunization, and weight monitoring of the baby; encourages the mother to participate in well-baby clinics, and counsels the mother regarding family planning [8].

The PHM is a pivotal field health worker who intervenes to improve maternal and child health. Assessing the postnatal services provided by PHMs in an equity sensitive manner helps to identify the gaps in home-based care which are unjustifiable. This study was conducted to determine the overall and sector-wise socioeconomic inequality in postnatal home visits made by public health midwives in Sri Lanka and to decompose the observed socioeconomic inequality into potential determinants.

## Materials and methods

### Data

Data from the Sri Lanka Demographic and Health Survey (SLDHS) 2006–07 were used. SLDHS was carried out in 20 districts of Sri Lanka, excluding the five districts of the Northern province due to the security situation that prevailed in those areas during the survey. A stratified two-stage cluster sampling design was used to recruit a nationally representative sample. The first stage involved selecting 2500 enumeration areas (clusters) from the list of about 100000 enumeration areas created for the 2001 Population Census. An enumeration area was considered a subdivision of a Grama Niladari area (smallest administrative unit in the country), which consists of about 80 housing units in urban areas and about 65 units in rural or estate (plantation) areas. Sri Lanka is divided into three sectors; urban, rural, and estate considering the geographical location and the availability of infrastructure facilities [13]. The second stage of selection involved the systematic sampling of 10 households listed in each enumeration area. Two thousand one hundred and six clusters were sampled, from which, 21060 housing units were selected, and 19862 households were interviewed. All ever-married women aged 15–49 years living in these households were eligible to be interviewed [9]. The dataset obtained from the Department of Census and Statistics included 14909 ever-married women.

The questionnaire used in the 2006–07 SLDHS collected information on household and women and children through personal interviews. In the household section, demographic information of the household members and the characteristics of the household dwelling were collected. The section for ever-married women collected information on their background characteristics, information related to sexual and reproductive health, child immunization and health, child and women’s nutrition, working status of women, use of drugs, tobacco, and alcohol by household members, other health issues, and husband/partner’s background characteristics [9].

#### Health variable (outcome) of interest

Whether the PHM visited the home at least once to provide postnatal care within one month of the delivery after giving birth to their last child was the health variable of interest. The response was dichotomous (yes/ no). SLDHS 2006–07 collected data from ever-married women who gave birth in 2001 or later (up to 2007). 6976 ever-married women had reported their last birth between 2001 and 2007. Among them, there was no information on the health variable of interest for 2049 women in the 2006–07 SLDHS dataset that we received. 4927 women had data for this question and 34 cases appeared to be marked as missing yielding 4893 ever-married women for the analysis.

### Data analyses

Sociodemographic characteristics of the sample were described using mean (SD), frequencies, and percentages. Percentage of the women who received a postnatal home visit by a PHM was reported across each level of sociodemographic characteristic. Pearson chi-square test was used to examine the association between each sociodemographic characteristic and receipt of at least one postnatal home visit by a PHM within one month of the delivery.

#### Measuring socioeconomic status

A proxy measure (wealth index) was constructed using the household ownership of durable goods and housing characteristics [14, 15]. Principal components analysis (PCA) was performed to calculate the wealth index. The variables used in the PCA were household ownership of durable consumer items such as a watch, a radio, a television, a mobile telephone, a land telephone, and a refrigerator; ownership of a bicycle, a motorcycle or a motor scooter, a three wheeled vehicle, a tractor or a land master, a motor car/van/bus/lorry, and, a boat with a motor; dwelling characteristics like material used for the floor, wall, and roof; persons per sleeping room; access to utilities and infrastructure facilities like source of drinking water, electricity, toilet facility, and type of cooking fuel used.

#### Measuring socioeconomic inequality

The concentration index (CI), derived from the concentration curve, was used to quantify the degree of socioeconomic inequality [16]. The concentration index is defined as twice the area between the concentration curve and the line of equality (the 45-degree line). A concentration curve plots the cumulative percentages of the health variable (y-axis) against the cumulative percentage of the population, ranked by living standards, beginning with the poorest and ending with the richest (x-axis) [14].

CI is expressed as[16]:

$$C=\frac{2}{n\mu }\sum_{i=1}^{n}y_{i}R_{i}-1$$ (1)

where, *C* is the CI, *n* denotes the number of observations (or individuals), *μ* is the overall mean of the health variable y, *y*_*i*_ is the health variable of interest of the i^th^ individual, and *R*_*i*_ is the rank of the i^th^ individual in the socioeconomic distribution moving from the most disadvantaged (poorest) to the least disadvantaged (richest) (Eq 1).

The sign of the CI indicates the direction of any relationship between the health variable of interest and the position in the living standard distribution. Its magnitude reflects both the strength of the relationship and the degree of variability in the health variable. The value of CI varies between -1 to +1 with 0 indicating no inequality. By convention, the CI is negative when the curve lies above the line of equality, indicating disproportionate concentration of the health-related variable amongst the poor and vice versa.

Socioeconomic inequalities of postnatal home visits made by PHMs within one month of delivery were estimated using concentration index and illustrated with concentration curves for the entire country and by the sector of residence (urban, rural, and estate).

#### Decomposition of the concentration index

As demonstrated by Wagstaff, van Doorslaer and Watanabe,[17] CI can be decomposed assuming a linear regression model for the health variable (y_i_) as:

$$y_{i}=\alpha +\;\sum_{k}\beta_{k}x_{ki}+\varepsilon_{i}$$ (2)

where, i is the i^th^ individual, *x*_*k*_ are set of determinants (explanatory variables). *β*_*k*_ denotes linear regression coefficient of determinant *x*_*k*_, and ε_i_ is the random error term for the i^th^ individual.

For any linearly additive regression model of the health variable of interest (y_i_) such as Eq 2 above, the CI for y can be written as:

$$C_{y}=\sum_{k}\left(\frac{\beta_{k}{\overset{-}{x}}_{k}}{\mu }\right)C_{k}+\frac{GC_{\varepsilon }}{\mu }$$ (3)

where, *C*_*y*_ is concentration index of y, *β*_*k*_ is the linear regression coefficient of determinant *x*_*k*_, $\overset{-}{x_{k}}$ is the mean of the determinant *x*_*k*_, μ is the overall mean of the health variable (y_i_), *C*_*k*_ is the concentration index for determinant *x*_*k*_, and GC_ε_ is residual component that captures income related inequality in the health variable that is not accounted for by systematic variation in determinants across socioeconomic groups. Eq 3 expresses the overall inequality of the health-related variable with two components including the deterministic or explained component and the unexplained component. The absolute contribution of each determinant to the total inequality is quantified by multiplying the health-related variable’s elasticity of that determinant $\left(\frac{\beta_{k}{\overset{-}{x}}_{k}}{\mu }\right)$ and its concentration index (*C*_*k*_). The relative percentage contribution of each determinant was calculated by dividing its absolute contribution by the CI of the health-related variable $(\frac{\beta_{k}x_{k}}{\mu })C_{k}/C$.

The above decomposition method was introduced to be used with linear, additively separable models [17]. As health-related variables are mostly dichotomous, multivariable analysis using non-linear estimation methods is required. The most common choices yielding probabilities in the range (0, 1) are the logit model and probit model, both of which are fitted by maximum likelihood.

$$y_{i}=\alpha^{m}+\sum_{k}{\beta_{k}^{m}x}_{ki}+\;u_{i}$$ (4)

One possibility when dealing with a discrete change from 0 to 1 is to use marginal or partial effects (dy/dx) which gives the change in the predicted probability associated with unit change in an explanatory variable [18]. An approximation of the non-linear relationship using marginal effects approximately restores the mechanism of the decomposition framework in Eq 3 through Eq 4. Therefore linear approximation of the non-linear estimation is given by Eq 4, where the $\beta_{k}^{m}$ are the marginal effects of each determinant *x*_*k*_ and *u*_*i*_ indicates the error generated by the linear approximation used to obtain the marginal effects [18]. Marginal effects demonstrate associations between the determinants and the health variable of interest, i.e. the probability of a health variable of interest occurring across each determinant. Positive signs are indicative of positive associations whilst those with negative signs indicate negative associations. The larger the absolute value of a marginal effect, the more substantial the association.

Hence, CI for a dichotomous health variable can be written with marginal effects as in (Eq 5):

$$C_{y}=\sum_{k}\left(\frac{\beta_{k}^{m}{\overset{-}{x}}_{k}}{\mu }\right)C_{k}+\frac{GC_{\varepsilon }}{\mu }$$ (5)

where, *C*_*y*_ is concentration index of y, $\beta_{k}^{m}$ is the marginal effects of determinant *x*_*k*_, $\overset{-}{x_{k}}$ is the mean of the determinant *x*_*k*_, μ is overall mean of the health variable (y_i_), *C*_*k*_ is the concentration index for determinant *x*_*k*_, and GC_ε_ is residual component.

We performed the decomposition analysis using probit regression. The explanatory variables used in the decomposition analysis were education level of women, ethnicity, education level of husband/partner, sector of residence, province of residence, and the wealth quintile. These covariates were selected a priori, in line with similar studies available in the literature [19–22]. Also, these covariates are recognised as social determinants of health that are closely linked with both health inequalities and inequities [23]. We did not have information on the age of the respondent at the last delivery but, respondent’s age at last birthday was available. We therefore did not include age as an explanatory variable in this study. All statistical analyses were performed using Stata version 12.

## Results

### Sociodemographic characteristics of the study participants

The mean (SD) age of the participants (n = 4890) was 30.2 (6.2) years. The majority of participants belonged to Sinhalese ethnicity living in the rural sector. More than 80.0% of women had completed secondary education or above. The education level of women, ethnicity, education level of the husband/partner, sector of residence, province of residence, and wealth index were associated with receipt of a postnatal home visit by a PHM within one month of the delivery. Respondent’s age at the time of data collection was not associated with the health outcome of interest (Table 1).

**Table 1:** Percentage of mothers received a postnatal home visit by a PHM within one month of delivery by sociodemographic characteristics.

Characteristic	Frequency (%)	Percentage of mothers received a postnatal home visit by a PHM^a^	p value^b^
**Age at last birthday (in years)**			
15–24	943 (19.3)	80.9	0.112
25–34	2721 (55.6)	83.8	
35–44	1170 (23.9)	81.3	
≥45	56 (1.2)	82.1	
**Education level of women**			
No education	146 (3.0)	68.5	<0.001
Primary	558 (11.4)	68.3	
Secondary	2700 (55.2)	84.2	
Higher or Degree	1489 (30.4)	86.6	
**Ethnicity**			
Sinhalese	3546 (72.5)	90.6	<0.001
Sri Lankan Tamil	396 (8.1)	71.2	
Indian Tamil	282 (5.8)	69.5	
Moor	648 (13.2)	51.5	
Other	21 (0.4)	85.7	
**Education level of husband/partner**			
Unaware	11 (0.2)	63.6	
No education	149 (3.0)	67.8	<0.001
Primary	774 (15.8)	74.2	
Secondary	2557 (52.3)	85.5	
Higher or Degree	1402 (28.7)	83.9	
**Sector of residence**			
Urban	915 (18.7)	80.1	<0.001
Rural	3550 (72.5)	84.5	
Estate	428 (8.8)	72.4	
**Province of residence**			
Western	1298 (26.5)	87.9	<0.001
Central	644 (13.1)	83.4	
South	620 (12.7)	91.5	
Eastern	576 (11.8)	47.4	
North Western	532 (10.9)	88.9	
North Central	341 (7.0)	88.6	
Uva	455 (9.3)	85.1	
Sabaragamuwa	427 (8.7)	85.0	
**Wealth index (quintiles)**			
Lowest	1153 (23.6)	80.0	<0.001
Second	1082 (22.1)	80.6	
Middle	960 (19.6)	85.0	
Fourth	899 (18.4)	87.3	
Highest	799 (16.3)	81.1	

^a^ percentage of mothers received a postnatal home visit by a PHM within one month of delivery for each category

^b^Pearson chi-square test for independence

### Socioeconomic inequality

**Fig 1:**
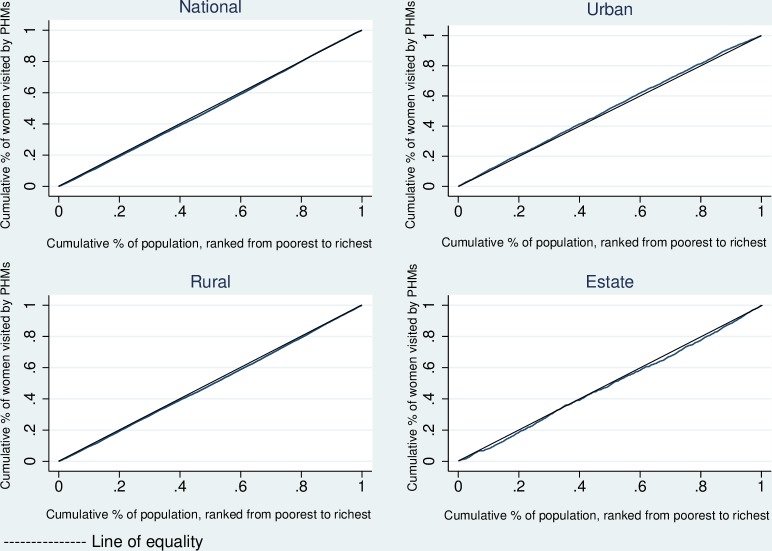
Overall and sector-wise concentration curves for home visits made by PHMs during the postnatal period.

Overall, 82.6% of women were visited by PHMs at least once during the first month after delivery. The highest number of home visits was reported in the rural sector (84.5%) and the lowest was reported from the estate sector (72.4%). The overall (national) and rural sector concentration indices were positive and significant though the absolute size was relatively small (Table 2). This indicates that the economically better-off women received more home-based care than those who were economically worse-off, a pro-rich inequality. In contrast, economically worse-off women in the urban sector received home-based care more than those who were economically better-off (Fig 1) implying a pro-poor inequality in the urban sector. No significant income related inequality in postnatal visits made by PHMs was observed in the estate sector.

**Table 2:** Overall and sector-wise socioeconomic inequalities of postnatal home visits made by PHMs within one month of delivery.

Sector	Number of women	Percentage of mothers received a postnatal home visit by a PHM within one month of delivery	Concentration Index (CI)	Standard Error (CI)	p value^b^
**National**	4893	82.6	0.011	0.003	0.004
**Urban**	915	80.1	-0.023	0.009	0.012
**Rural**	3550	84.5	0.014	0.004	0.001
**Estate**	428	72.4	0.026	0.017	0.131

^b^one sample t-test

### Decomposition of the socioeconomic inequality

**Fig 2:**
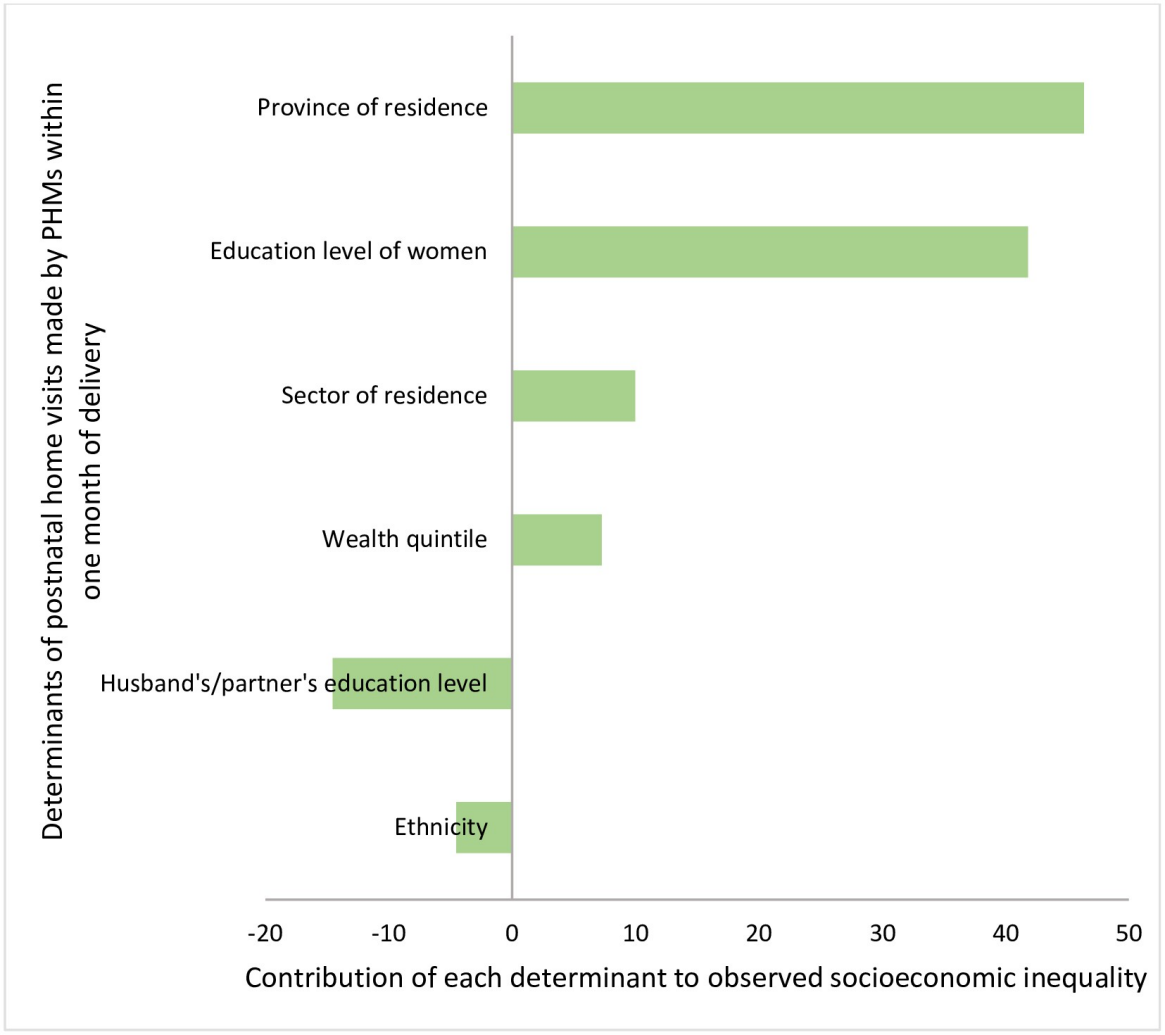
Contribution of each determinant to the observed socioeconomic inequality in postnatal home visits made by PHMs.

As stated earlier, the overall postnatal visits made by PHMs within one month of the delivery were more concentrated among economically better-off women (CI = 0.011). Province of residence was the main contributory factor for the observed socioeconomic inequality followed by education level of women, sector of residence, and wealth index (Fig 2). Interestingly, education level of the partner negatively contributed to the inequality mainly due to increased probability of the health outcome (positive marginal effects) across education categories where the poor people were disproportionately concentrated (negative concentration indices) (Table 3).

**Table 3:** Decomposition of observed socioeconomic inequality of postnatal home visits made by PHMs within one month of delivery.

Determinants	Marginal effect $\left(\beta_{k}^{m}\right)$	Weighted mean of the determinant ${(\overset{-}{x}}_{k})$	Concentration index of the determinant (*C*_*k*_)	Contributions to overall CI (CI = 0.011)	
				Absolute^‡^	Relative (%)^†^
**Education level of women *(Ref*: *No education)***					
Primary	-0.0323	0.1041	-0.3839	0.0015	
Secondary	0.0220	0.5537	-0.0910	-0.0013	
Higher or Degree	0.0350	0.3140	0.3382	0.0044	
			***Sub total***	**0.0046**	**41.82**
**Ethnic group *(Ref*: *Other ethnic groups)***					
Sinhalese	0.0245	0.7655	0.0210	0.0004	
SL Tamil	-0.0351	0.0651	-0.1487	0.0004	
Indian Tamil	-0.0842	0.0413	-0.5080	0.0021	
Moor	-0.2222*	0.1246	0.1025	-0.0034	
			***Sub total***	**-0.0005**	**-4.55**
**Education level of husband/partner *(Ref*: *Unaware)***					
No Education	0.0411	0.0290	-0.4736	-0.0006	
Primary	0.0491	0.1485	-0.3681	-0.0032	
Secondary	0.0923	0.5276	-0.0607	-0.0035	
Higher or Degree	0.0472	0.2928	0.3446	0.0057	
			***Sub total***	**-0.0016**	**-14.55**
**Sector of residence *(Ref*: *Estate)***					
Urban	0.0427	0.1175	0.3604	0.0021	
Rural	0.0662**	0.8276	-0.0152	-0.0010	
			***Sub total***	**0.0011**	**10.00**
**Province of residence *(Ref*: *North Central)***					
Western	0.0276	0.2640	0.3339	0.0029	
Central	0.0206	0.1326	-0.1648	-0.0005	
South	0.0464*	0.1265	-0.0181	-0.0001	
Eastern	-0.2191****	0.1185	-0.1034	0.0032	
North Western	0.0250	0.1085	-0.0950	-0.0003	
Uva	0.0047	0.0921	-0.2679	-0.0001	
Sabaragamuwa	0.0063	0.0870	-0.0943	-0.0000	
			***Sub total***	**0.0051**	**46.36**
**Wealth Quintile *(Ref*: *Lowest)***					
Second	-0.0076	0.2261	-0.3233	0.0006	
Middle	0.0216	0.2079	0.0826	0.0004	
Fourth	0.0286	0.1911	0.4772	0.0031	
Highest	-0.0216	0.1498	0.8606	-0.0033	
			***Sub total***	**0.0008**	**7.27**
**Explained**				**0.0095**	**86.4**
**Residual**				**0.0015**	**13.6**

**** p<0.001

*** p<0.01

** p<0.05

*p<0.1 (multivariable probit regression-Z-test)

Effective sample size = 4891 Weighted mean of the health variable (*μ*) = 0.8338

*Ref-*reference groups used in the probit regression

^‡^Absolute contribution of each determinant *x*_*k*_ to overall $CI=(\frac{\beta_{k}^{m}{\overset{-}{x}}_{k}}{\mu })C_{k}$

^†^Relative contribution to overall CI (%) = (Absolute contribution of each determinant to CI/0.011)*100

Significant positive marginal effects were observed for the rural sector and the Southern province whilst significant negative marginal effects were observed for the Moor ethnic group and for residents of the Eastern province (Table 3). After adjusting for all other variables, being a woman residing in the rural sector increased the probability of being visited by a PHM almost 6.6% compared with a woman residing in the estate sector. Being a Moor woman decreased the probability of being visited by a PHM by 22.0%. Being a woman living in the Eastern province also decreased the probability of being visited by a PHM by 22.0%. A positive gradient of marginal effects was observed with increased education level of women but this was not significant. A similar pattern was observed with the partner’s education level up to secondary education. The variables entered into this model explained 86.4% of the observed socioeconomic inequality in postnatal home visits made by PHMs within one month of delivery.

## Discussion

### Summary of main findings

Eighty three percent of women were visited by PHMs at least once within the first month of the delivery. Overall, we found a pro-rich inequality in postnatal visits made by public health midwives in Sri Lanka probably due to the majority of the Sri Lankan population being resident in the rural sector. However, the results varied by the sector of residence (urban, rural, and estate) indicating differences within and between sectors. The main contributory factors for socioeconomic inequality were province of residence and education level of women.

### Comparison with the existing literature

Our findings corroborate with the existing literature. Postnatal care was disproportionately concentrated among economically better-off women in Odisha, India[24], Nepal[25], and Nambia[26]. However, in our study, the pattern of socioeconomic inequality of postnatal home visits varied across sectors. Interestingly, postnatal visits were more concentrated among economically worse-off women in the urban sector implying that women who were in need had received care in the urban sector. This could be partly because affluent people seek private medical care. In contrast, postnatal home visits were disproportionately concentrated among economically better-off women in the rural sector. Similar results have been reported from Western rural China where a pro-rich inequality was found in receiving at least two postnatal visits with a CI of 0.084 [27]. In our study, the number of postnatal home visits made by PHMs in the estate sector (72.4%) was below the national figure and no income inequality was found across the sector. This could be due to the large proportion of economically worse-off people living in the estate sector. In 2006–2007, 32.0% of people in the estate sector were living below the poverty line [28]. Estate sector residents are predominantly descendants of migrant labourers from South India brought to the country during colonial rule. The successive governments have taken substantial measures to uplift their living conditions and absorb them into the mainstream. However, we still see the gaps in certain areas as country-wide interventions sometimes do not work with them [29].

In decomposition analysis, the contribution of a particular determinant to the observed socioeconomic inequality depends on two factors; 1) the impact of the determinant on the health variable of interest and 2) the degree of socioeconomic inequality in that factor (how unequally that determinant is distributed over socioeconomic strata). The partner’s education level negatively contributed to the observed socioeconomic inequality (Table 3). This implies that there is an increased probability of a PHM visiting a mother (positive marginal effect) across partner’s education categories. However, the concentration indices of each education category reflect that poor people were disproportionately concentrated in all education categories except in the ‘higher or degree’ category. The absolute contribution of each determinant to the total inequality is quantified by multiplying the health-related variable’s elasticity of that determinant $\left(\frac{\beta_{k}{\overset{-}{x}}_{k}}{\mu }\right)$ and its concentration index. When the health variable of interest is dichotomous, non-linear estimation methods are used; the linear regression coefficient *β*_*k*_ substitutes from the marginal effect of that determinant $\left(\beta_{k}^{m}\right)$. As the absolute contributions of the lowest three educational categories were negative, the total absolute contribution of partner’s education level was negative.

The main contributory factors for the observed socioeconomic inequality were province of residence and education level of women. A geographic disparity was observed in postnatal PHM visits. Being a woman residing in the Eastern province decreased the probability of being visited by PHMs. This lower probability corresponds with data of the Family Health Bureau which reported that only 40.0% of women in the Trincomalee district of the Eastern province had received a postnatal visit by a PHM within 10 days of delivery between 2004 and 2006 [30, 31]. This may have been due to the security situation in that area during this period.

The education level of the women was the second most contributing factor for the observed inequality. The likelihood of being visited by a PHM during the postnatal period was increased with increased education level of women. This finding is consistent with the results of a previous study [19]. Furthermore, the education level of women has been identified as a main factor facilitating the utilization of maternal health services globally [32–35] and a major determinant of inequality of maternal health services utilization [19, 24, 26] including the receipt of postnatal care [27]. Previous studies suggest that a better wealth status is associated with increased utilization of maternal health services as it enables women to afford the transport cost to reach the health care facility [36]. In Sri Lanka, women are not always required to reach the health facility by themselves to receive postnatal care; it is a combination of care provided through hospitals, field clinics, and home visits. The field PHM is supposed to visit postnatal mothers at home at different time intervals. This fact has been reflected in our decomposition analysis where wealth was only a small contributor to the observed socioeconomic inequality.

Ethnicity and religion are two inter-connected determinants that influence a person’s health seeking behaviour. In many parts of the world, cultural beliefs, norms, and values are particularly linked with uptake of maternal health services [33, 35]. Our decomposition analysis revealed that being a Moor woman decreased the probability of receiving postnatal home visits by PHMs. The majority of the population living in the Eastern province is of Sri Lankan Moor ethnicity. Previous research suggests that ethnic minority groups are less likely to use maternal health services compared with their counterparts [37, 38]. In rural China, ethnic majority women (Han) were more likely to use postnatal care [27]. In contrast, no significant association was found between ethnicity and use of postnatal care facilities in Ethiopia [32].

According to statistics of the Family Health Bureau, 85.3% (2005) and 90.1% (2007) Sri Lankan women reported at least one postnatal PHM visit during the first 10 days of the delivery [30, 31]. Approximately 20.0% had received at least one postnatal visit by a PHM between the 11^th^ and 28^th^ day after delivery. These figures suggest that we need further improvements in achieving the recommended number of postnatal visits by a PHM, a number of factors influence achieving this target. One is loss to follow up. A woman who has delivered a baby may leave her own PHM area and go to another place such as her parents’ home to spend the postnatal period. In Sri Lanka, a large number of women prefer to go to their own ancestral homes soon after the delivery to get help from their mothers. Another factor is not informing the PHM of the delivery in time. The PHM relies on different sources of information such as a formal notification from the hospital where the child was delivered, direct information from relatives, information from another PHM or her own records on the expected date of delivery. The excessive work load of PHMs may be associated with low coverage [11].

Analysis of monthly reports sent by PHMs in 2007 revealed that the average number of postnatal home visits made by PHMs was below the expected number. Heavy workload reported by PHMs may be significantly associated with low performance indicators. In seven out of nine provinces in Sri Lanka, it has been revealed that PHMs spend on average 48.7 hours per week carrying out their duties, and 3.7 hours in excess of their official duty hours. One third of their work time was spent on home visits and 81.0% of their time was spent on maternal and child care activities, some of which were done during home visits. The perceived work load and actual work hours of PHMs have increased with the increased population size as well as the additional functions that have been recently included in their duty list [39].

### Strengths and limitations of the study

Our study was based on the Demographic and Health Survey data of 2006–07 which included a large representative sample of Sri Lankan reproductive aged women. This is the first study of its kind which investigated the socioeconomic inequality in postnatal care in Sri Lanka. Demographic and Health Surveys (DHS) are nationally-representative household surveys that provide data in the areas of population, health, and nutrition. These surveys follow a rigorous methodology and several steps and have been taken to improve the quality of data of these surveys over time [9].

We used data from the 2006–07 SLDHS which was the latest one available at the time of analysis; subsequently a Demographic and Health Survey was carried out in 2016, the results of which were not published nor available when this study was carried out. Nonetheless, findings of this study are still useful to make comparisons over time to assess the effectiveness of new interventions in terms of reducing inequalities and inequities related to provision of postnatal care by Public Health Midwives. As the dataset used for this study is part of phase five of DHS surveys (conducted between 2003 and 2008), which includes information on standardized variables, cross-country comparisons of socioeconomic inequalities in postnatal care provision by PHMs are also possible.

The main outcome measure of this study, attendance of a PHM within one month of delivery, is likely to be subject to recall bias. Generally in Sri Lanka, mothers are encouraged to keep their pregnancy record until their next pregnancy. This record has a section for post-partum field care where PHM has to mention the date that she made the first home visit. The 2006–07 SLDHS dataset had a variable which recorded whether the interviewer had seen the pregnancy record of the respondent. Two-thirds of the participants (65.1%) have presented the pregnancy record to the interviewer, although there was an increased chronological gradient. Hence, we can presume that the responses of these women may not be greatly subjected to recall bias. A range of measures were taken to improve the quality of data. A calendar of events was used to record information related to respondent’s marriage, pregnancies and births, and contraceptive use in a specially designed chart for a five-year period prior to the survey. The entire questionnaire was pre-tested by a team of experienced staff to test the feasibility, sequence, skipping, and timing before it was finalised. The majority of the data collectors comprised staff of the Department of Census and Statistics, Sri Lanka who were experienced in conducting surveys [9].

The 2006–07 SLDHS excluded five districts of the Northern Province (Jaffna, Kilinochchi, Mannar, Vavuniya, and Mullativu) due to the security situation during the time of survey. Hence, this dataset only represents the eight Provinces of Sri Lanka which included 20 districts. The 2006–07 SLDHS dataset contained 6976 ever-married women who reported their last birth between 2001 and 2007. Of them only 4927 women (70.6%) had data for the health variable we were interested in. 2049 women had no information in the dataset and it was not marked as missing data either. Hence our findings need to be interpreted with caution.

We could not study the socioeconomic inequality of the recommended number of postnatal home visits made by PHMs within one month of the delivery. Future studies should address this aspect.

### Implications for public health practice

The magnitude of the socioeconomic inequality of postnatal home visits made by PHMs is very small in Sri Lanka. More importantly, the inequality observed in the urban sector was not unjust or unfair. Therefore, when measuring performance of health outcomes it is sensible to link these indicators with health inequality perspectives. However, as anticipated, the estate sector was lagging behind the other sectors. It appears that multi-sectoral collaborations are required to improve the social determinants of health affecting provision of postnatal care in Sri Lanka. Specific interventions should be implemented targeting working conditions and cultural elements in the estate sector [29].

According to the findings of this study, around 20.0% of women in Sri Lanka have reported that they were not visited by a PHM within one month of the delivery. This figure is even higher in the estate sector (around 30.0%). The standards set by the national maternal and child health programme stipulate that three postnatal home visits should be made by PHM within one month of delivery. Although 83.0% of women were visited by PHMs at least once within the first month of the delivery, the percentage of mothers who were visited three times within one month of delivery is likely to be much less. This raises questions regarding the quality of recommended care received by postnatal mothers. We understand that certain factors affecting this performance indicator (loss to follow up, not informing the delivery to PHM in time) are sometimes beyond the control of PHMs. However, we urge Public Health Midwives, irrespective of their sector of work, to make the recommended number of post-natal home visits according to national guidelines, making it as a priority in their duty list.

We also recommend carrying out equality analysis of health care performance indicators to set fair priorities and for effective resource allocation. It is important to identify and address broad social determinants of health related to specific health issues. Another application of inequality analysis is tracking health inequalities over time. It helps to understand the effect of new health interventions or policy measures in terms of reducing health inequalities as well as inequities [22, 40, 41]. Sri Lankan health authorities should incorporate health inequality measures into existing health indicators to track progress over time in reducing inequalities.

## Conclusion

A pro-rich inequality was found in postnatal home visits made by public health midwives in Sri Lanka. The magnitude of the socioeconomic inequality was very small. However, the results varied by the sector of residence (urban, rural, and estate). Province of residence and the education level of women were the main determinants of the observed socioeconomic inequality.
